# P-1022. Ventricular Shunt Failure in Patients with Coccidioidal Meningitis in Central California, A Single Center Experience

**DOI:** 10.1093/ofid/ofae631.1212

**Published:** 2025-01-29

**Authors:** Elise Hyser, Geetha Sivasubramanian, Kenneth Fox, Alyssa Hughes, Jasmine Sandhu, Yueqi Yan, Pavel Diaz

**Affiliations:** University of California San Francisco-Fresno, Fresno, California; UCSF Fresno, Fresno, California; UCSF School of Medicine, San Francisco, California; University of California San Francisco-Fresno, Fresno, California; Clovis East High School, Fresno, California; UC Merced, merced, California; University of California Merced, Merced, California

## Abstract

**Background:**

*Coccidioidal* meningitis (CM) represents the most deleterious form of disseminated coccidioidomycosis and warrants lifelong antifungal therapy. Communicating hydrocephalus is a well-known complication of CM. Antifungal therapy and lumbar drain placement is typically insufficient treatment for CM-related hydrocephalus, often requiring shunt placement. Shunt failures and repetitive surgical revisions have been described in patients with CM. We aimed to characterize shunt outcomes in patients with CM with ventricular shunts in our center.

**Figure:**
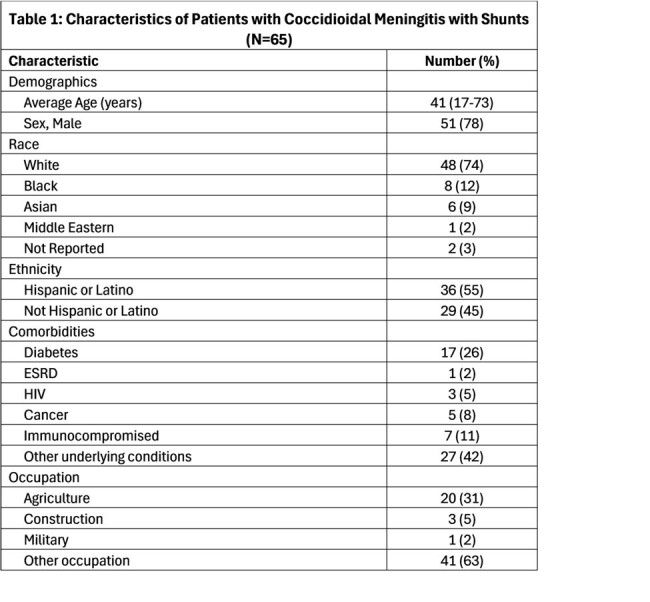

**Methods:**

We performed a retrospective study of patients above the age of 18 with CM requiring shunt placement in our center from 2014-2024. Data pertaining to demographics, comorbidities, clinical presentation, laboratory and imaging data, shunt characteristics, management and outcomes were collected. Descriptive statistics were used to analyze the sample characteristics. Fisher’s exact or Chi-square test was used to compare outcomes using categorical variables and T-test for continuous variables.

**Figure:**
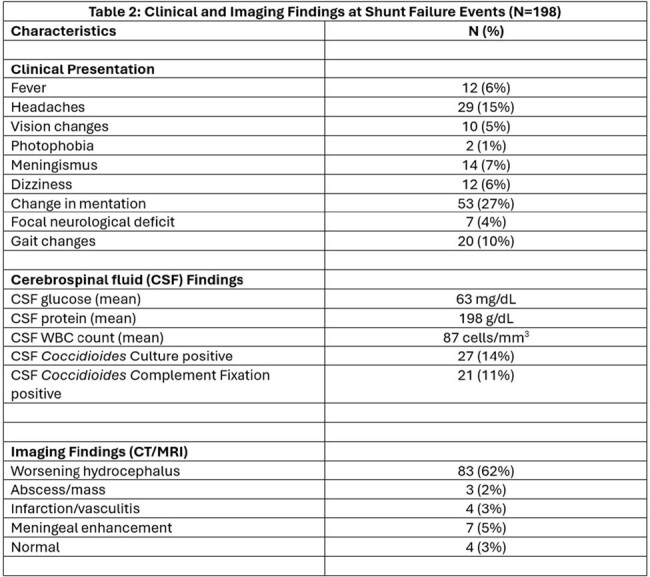

**Results:**

We identified 65 patients with CM and shunt placement. Of these, 27 (42%) did not have shunt failure while 38 (58%) had one or more shunt failure events (ranging 1-14 times, total of 198 events in the whole cohort). Mean age of patients in was 41 years, 55% were Hispanic and 38% had outdoor occupational exposure (Table 1). The average number of days between shunt placement and initial shunt failure was 46 days (0-174). Change in mentation was noted in patients during 27% of shunt failure events (Table 2). Infections and shunt malfunction were major reasons for shunt failure (Figure 1). Patients that suffered shunt failure events, had a statistically significant higher risk of death (p value 0.01).

**Figure:**
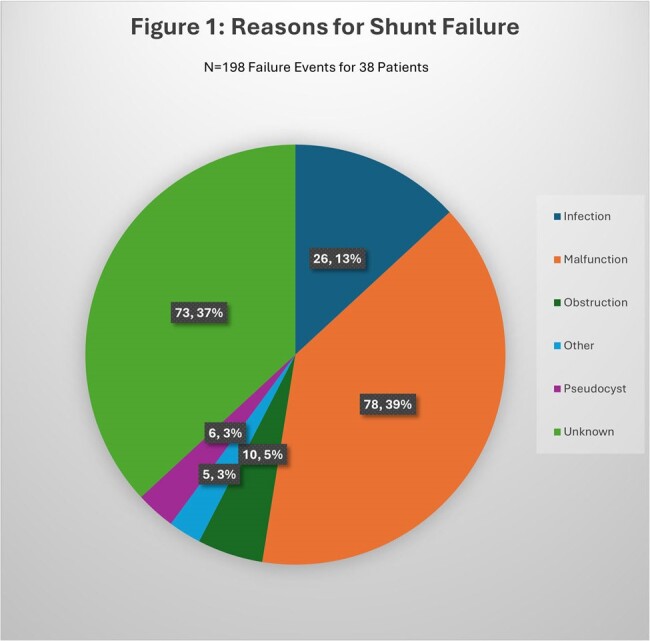

**Conclusion:**

CM related hydrocephalus, a common complication of this infection, remains a difficult condition to manage. Even with availability of potent anti-fungal agents, 58% of our patients developed shunt failure needing repeated hospitalizations and shunt revision surgeries. Patients who developed shunt failure in our cohort had a higher risk of death. More studies are needed to identify modifiable risk factors which may help prevent this complication and further reduce morbidity and mortality.

**Disclosures:**

**All Authors**: No reported disclosures

